# *Editorial Commentary:* Host and Viral Factors in Emergent Influenza Virus Infections

**DOI:** 10.1093/cid/ciu054

**Published:** 2014-01-31

**Authors:** David S. Hui, Frederick G. Hayden

**Affiliations:** 1 Department of Medicine and Therapeutics, Chinese University of Hong Kong, Shatin; 2 Division of Infectious Diseases and International Health, Department of Medicine, University of Virginia School of Medicine, Charlottesville

**Keywords:** influenza, avian influenza, virulence, host genetics, smoking

**(See the Major Article by Wang et al on pages 1095–103.)**

Human cases of avian influenza A(H5N1) virus infection were first documented in Hong Kong in 1997 [1, 2]; the virus reemerged in 2003 to spread intercontinentally and become entrenched in poultry in other parts of Asia and Egypt. Sporadic human cases with an estimated case fatality rate close to 60% continue to occur [3]. Influenza A(H1N1)pdm09 virus first emerged in 2009 as a novel swine-origin strain that rapidly led to a pandemic [4] and remains a common circulating strain. Human infections with the novel avian influenza A(H7N9) virus, first reported in China in March 2013 and subsequently declining after closure of live poultry markets, have reemerged since October 2013 in mainland China and spread to Hong Kong in December 2013 [5]. All 3 types of influenza viruses continue to pose significant threats to human health globally.

The retrospective cohort study published by Wang et al in this issue of *Clinical Infectious Diseases* [6] compares the demographic features, risk factors, presenting clinical characteristics, and outcomes of patients hospitalized in China and Vietnam with laboratory-confirmed avian A(H7N9), avian A(H5N1), or A(H1N1)pdm09 virus infection. The authors have combined and compared data derived from selected studies with somewhat differing designs and time frames. For example, data from Vietnamese patients with A(H1N1)pdm09 or with A(H5N1) infection from April 2009 onward are not included [6], and population-based data on baseline comorbidities were not available for Vietnam. Such disparities might have resulted in populations with differences in variables of interest being combined or missing for analysis. Secular changes in case management are also potential confounders with regard to outcomes such as intensive care unit admission and mortality. In the current report, the frequency of mechanical ventilation was slightly higher in patients with A(H7N9), all of whom were admitted within the past year, than in A(H5N1) patients, whereas the case fatality rate was substantially higher for A(H5N1).

The analysis of risk factors compares their proportions in the general Chinese population to those in the hospitalized influenza patients. The findings confirm prior studies highlighting the older age and male predominance of A(H7N9) patients [7], the broad similarities in clinical and laboratory features of severely ill A(H7N9) and A(H5N1) patients [8, 9], and the findings that obesity, asthma, and chronic obstructive pulmonary disease are significant risk factors in hospitalization for A(H1N1)pdm09 virus infections [4]. The finding that chronic heart disease was associated with an increased risk of A(H7N9) hospitalization is unsurprising, given the older age of these patients and chronic heart disease's strong association with seasonal influenza complications, although only 11% of patients with A(H7N9) reportedly had chronic heart disease [6]. Recent retrospective analyses examining the effects of influenza-specific interventions have highlighted the importance of influenza infections as predisposing to serious cardiovascular events. Both seasonal influenza vaccine receipt [10] and neuraminidase inhibitor treatment for clinically diagnosed influenza [11, 12] have been associated with significant reductions in subsequent acute cardiac and stroke diagnoses.

Both viral and host factors contribute to disease severity and outcomes across influenza subtypes. The younger age, infrequent presence of comorbidities (11% of cases), and higher mortality of A(H5N1) patients (55%) presumably reflects the greater intrinsic virulence of A(H5N1) viruses, although these viruses also show substantial genetic diversity and differences in virulence and treatment response in ferrets [13] and perhaps in patients [14]. Although the absolute risk of severe disease is thankfully much lower for A(H1N1)pdm09 infection compared with A(H5N1) and A(H7N9), younger age was also a risk factor for increased likelihood of hospitalization and mortality, relative to seasonal influenza, during the first year of the pandemic [15]. Of note, the pattern of excess deaths in those aged <65 years may continue up to a decade after introduction of a pandemic strain [16], consistent with recent reports of severe A(H1N1)pdm09 virus infections in the United States (http://emergency.cdc.gov/HAN/han00359.asp) and elsewhere.

One counterintuitive observation in the current report was an apparent protective effect of smoking on hospitalization across all 3 virus subtype cohorts. One possible hypothesis is that nicotine-related anti-inflammatory effects might alter influenza severity in humans. Chronic infusion of nicotine at doses chosen to model plasma levels of those found in smokers has been associated with reduced lung inflammation and increased survival, but also increased viral replication in influenza-infected mice [17]. In contrast, smoking has been linked to increased influenza severity in most earlier epidemiologic studies [18–20], although not in all [21], and also in reports from the 2009 pandemic [22–24]. In one murine model of influenza, chronic cigarette smoke exposure was associated with higher lung viral loads and worse disease, in part mediated by reductions in pulmonary T-cell interferon-γ production [25]. Another study found greater pulmonary inflammation and mortality, although not viral replication, in influenza-infected mice given short-term exposures to cigarette smoke compared with nonexposed mice [26]. Smoking is a risk factor associated with community-acquired pneumonia [20], underlying cardiopulmonary conditions such as chronic bronchitis, and often with male sex, which may account in part for the somewhat higher prevalence (25%) of smoking among the A(H7N9) patients compared to the A(H5N1) and A(H1N1)pdm09 patients in the current report [6]. Although further data on the interactions of tobacco smoke, its constituent components, and its associated health consequences with influenza infection are needed, smoking cessation remains a healthcare priority for many reasons.

Another key issue, not addressed in the current paper, is the presence of host genetic factors that may be related to influenza disease susceptibility and/or severity [27]. One specific allele in the interferon-induced transmembrane 3 (*IFITM3*) gene has been linked to increased severity of A(H1N1)pdm09 virus infections [28]. An estimated 25% of the Han Chinese population is homozygous for this allele. The CC genotype of the single-nucleotide polymorphism (SNP) rs12252-C allele has been found in 69% of Chinese patients with severe influenza A(H1N1)pdm09 virus infection compared with 25% in those with mild infection. Specifically, the CC genotype was estimated to confer a 6-fold greater risk for severe infection than the CT and TT genotypes [29]. Interestingly, Wang et al have identified the rs12252-C genotype as a primary genetic correlate of severe A(H7N9) pneumonia and excessive proinflammatory mediator blood levels [30]. Another recent study has shown that an allele of rs1130866, a SNP in the surfactant protein B gene (*SFTPB*), was associated with severe influenza A(H1N1)pdm09 infection in patients in Hong Kong in whom the CC genotype was overrepresented in comparison to the general Han Chinese population (odds ratio = 3.232) [31]. The carriage frequencies of alleles related to human leukocyte antigen binding efficiencies to influenza epitopes and T-cell responses have also been linked to A(H1N1)pdm09 virus mortality [32]. The frequency of a 32-bp deletion in the CCR5 gene (CCR5Δ32) was reported as unexpectedly high at 56% in Caucasian patients with critical illness caused by influenza A(H1N1)pdm09 [33]. The strong familial clustering of A(H5N1) cases among blood relatives also suggests underlying host genetic susceptibility [34]. Studies of sufficiently large numbers and clearly defined phenotypes such as severe viral pneumonia will likely find other host genetic determinants that contribute to influenza severity and potentially might lead to new therapeutic options.

In addition to age and presence of comorbidities, other factors including the type of virus exposure and initial inoculum size, frequency of bacterial coinfections, and timely disease recognition and access to quality care are likely important factors in affecting both clinical presentation and ultimate outcome in influenza. Retrospective analyses show that the time from symptom onset to antiviral treatment had a substantial impact on mortality in hospitalized A(H1N1)pdm09 patients in China [35] and elsewhere and also in A(H5N1) patients [14]. In this regard, the emergence of oseltamivir resistance during therapy in some critically ill patients has been linked to poor outcomes during A(H5N1), A(H1N1)pdm09, and, recently, A(H7N9) infections [36]. Access to high-quality critical care is certainly essential to survival, but some interventions might contribute to worse outcomes. Retrospective analyses suggest that the administration of systemic corticosteroids, a commonly used intervention given for influenza-associated pneumonia and acute respiratory distress syndrome, appears to be associated with increased risks of secondary infections and mortality [37].

In summary, A(H5N1), A(H1N1)pdm09, and A(H7N9) continue to be the major influenza infections of global concern. This report also reminds us of the health threats posed by other emergent influenza viruses, again reinforced by the recent finding of a lethal avian H10N8 subtype infection in an older Chinese woman [38, 39]. Careful analysis of those affected by emergent influenza viruses, as in the report by Wang et al [6], is essential to improving risk assessment and case detection and management.
